# A wait-and-watch approach to small pancreatic neuroendocrine tumors: prognosis and survival

**DOI:** 10.18632/oncotarget.7902

**Published:** 2016-03-03

**Authors:** Sara Massironi, Roberta Elisa Rossi, Alessandra Zilli, Giovanni Casazza, Clorinda Ciafardini, Dario Conte

**Affiliations:** ^1^ Gastroenterology and Endoscopy Unit, Fondazione IRCCS Ca' Granda Ospedale Maggiore Policlinico, Milan, Italy; ^2^ Postgraduate School of Gastroenterology, Department of Pathophysiology and Transplantation, Università degli Studi di Milano, Milan, Italy; ^3^ Department of Biomedical and Clinical Sciences “L. Sacco”, University of Milan, Milan, Italy

**Keywords:** pancreatic neuroendocrine neoplasms, neuroendocrine tumors, pNEN, non-functional pancreatic neuroendocrine tumors

## Abstract

**Background:**

Whether all the small (ø≤20mm) non-functional pancreatic neuroendocrine neoplasms (pNENs) should be routinely resected is unclear.

**Aim:**

To assess the overall survival (OS) and progression-free survival (PFS) of patients with small pNENs, followed-up with different management options.

**Material and methods:**

Between 2007-2014, 51 patients were newly diagnosed with pNEN. 15 patients with pNENs ø ≤20 mm underwent an intensive follow-up at 3-month intervals during the first year and then every 6 months (FU pNEN group). They were all at TNM stage I, except for one patient at stage IIA. 21 patients underwent surgical resection (SR pNEN group): 2 patients were at TNM stage I, 9 IIA, one IIIB, 9 IV. 15 patients received systemic therapy (ST pNEN group) due to advanced disease or contraindications to surgery: 5 were at stage IIA, 2 IIB, 8 IV.

**Results:**

The median follow-up for the entire cohort was 50 months. Survival was similar in the FU and SR pNEN groups, but significantly worst in the ST pNEN patients (log-rank test P <0.05). The 4-year survival rate was 100% in the FU pNEN group, 90.5% among the SR pNEN patients, 61% for the ST pNEN ones (p <0.0001). The disease remained stable in all but one patient in the FU pNEN group, whereas six patients in the SR group and five in the ST group showed disease progression.

**Conclusions:**

The “wait-and-watch” approach to early-stage small pNENs appears to be safe although further studies are needed to confirm these results in larger cohorts of patients.

## INTRODUCTION

Pancreatic neuroendocrine neoplasms (pNENs) are rare neoplasms, even if their incidence is on the increase worldwide [1–3] as well documented over the last two decades [4]. As recently pointed out by SEER the incidence of pNENs with a ≤ 2 cm size has increased by 710.4% (with an annual 12.8% change) over 22 years [5]. Such a fact may result from the increasing use of endoscopic ultrasound, which implies the greater ability to detect small pNENs.

PNENs represent ca. 2% of all pancreatic tumors and may be either non-functioning (50% to 90%) or functioning (10% to 50%). PNENs are characterized by great biological variability. It has been hypothesized that larger neoplasms have a greater potential for aggressive behavior, whereas smaller (ø ≤2 cm), low-grade, non-functioning tumors usually display a more benign behavior, with slow growth and an overall good prognosis [5]. However, nodal and distant metastases as well as disease recurrence have been documented with regard to small tumors, suggesting that such tumors too may have a malignant potential [5–9]. On the other hand, good overall survival (up to 100% after 5 years) and little impact on survival from small pNENs, incidentally discovered, have been described [10–11]. In a recent bi-institutional study Gaujoux et al. [12] observed that none of their 46 patients with small incidentally discovered pNENs had developed distant or nodal metastases after a median follow-up of 34 months and an average number of serial imaging sessions at 4. In 6 patients (13%) a ≥20% increase in size was observed, but no patient nor tumor characteristics were found to be significant predictors of tumor growth.

Several attempts to determine factors that are predictive of tumor growth, nodal or distant metastases, or survival have been made, with inconsistent results until now.

With these areas of uncertainty in mind, surgical resection has always been considered as the most appropriate management option for these tumors because of: their heterogeneous, often unpredictable biological behavior, the lack of specific prognostic factors and data from the literature suggesting a positive effect of surgery on overall pNEN survival. In a retrospective study on 380 patients Sharpe et al. [13] demonstrated that with regard to non-functional ø ≤2 cm sized pNENs surgical resection delivers a survival benefit, with a five-year overall survival (OS) of 82.2% for patients who underwent surgery and 34.3% for patients who underwent observation only. Noteworthy, the tumor size and margin status were not predictors of survival, whilst lymph node positivity was found to be associated with a decreased 5-year OS. On the other hand, a relevant morbidity (<5%) and mortality (40%–50%) is associated with pancreatic resection [14–15] and long-term exocrine and endocrine pancreatic insufficiency may affect the quality of life [16].

Despite a substantial controversy regarding the best management options for pNENs smaller than 2 cm, the European Neuroendocrine Tumor Society (ENETS) guidelines now recommend a “wait and see” policy in selected patients with small asymptomatic pNENs [17] in view of the slow growth of such tumors and good overall survival [10]. This strategy has gained acceptance for multiple endocrine neoplasia type 1 (MEN-1) patients [18–20] and some retrospective series have validated this approach also with regard to sporadic incidentally identified non-functioning pNENs smaller than 2 cm. Lee et al. [21] observed a nearly 50% risk of complications among the 57 patients who underwent surgery. The remaining 77 patients were conservatively managed and they were all free of disease progression, suggesting that non-operative management may be advocated for carefully selected pNENs when serial imaging demonstrates minimal or no growth without suspicious features.

However, the currently recommended conservative approach to small pNENs is based on under-powered and retrospective series and the resection of small, non-functioning tumors has not been compared to date with the conservative approach in prospective trials.

Based on the above reported findings, our present prospective series was aimed at evaluating both OS and progression-free survival (PFS) of patients with small pNENs evaluated at a single Institution according to the different clinical management options.

## RESULTS

The median follow-up for the entire cohort was 50 months (range 2-84). The overall 4-year survival rate was 86%. At the end of the study, 44 patients (86%) were still alive. Of the 7 patients (14%) who had died, 6 died of disease-related causes, whereas one patient passed away for unrelated causes.

**Figure 1:**
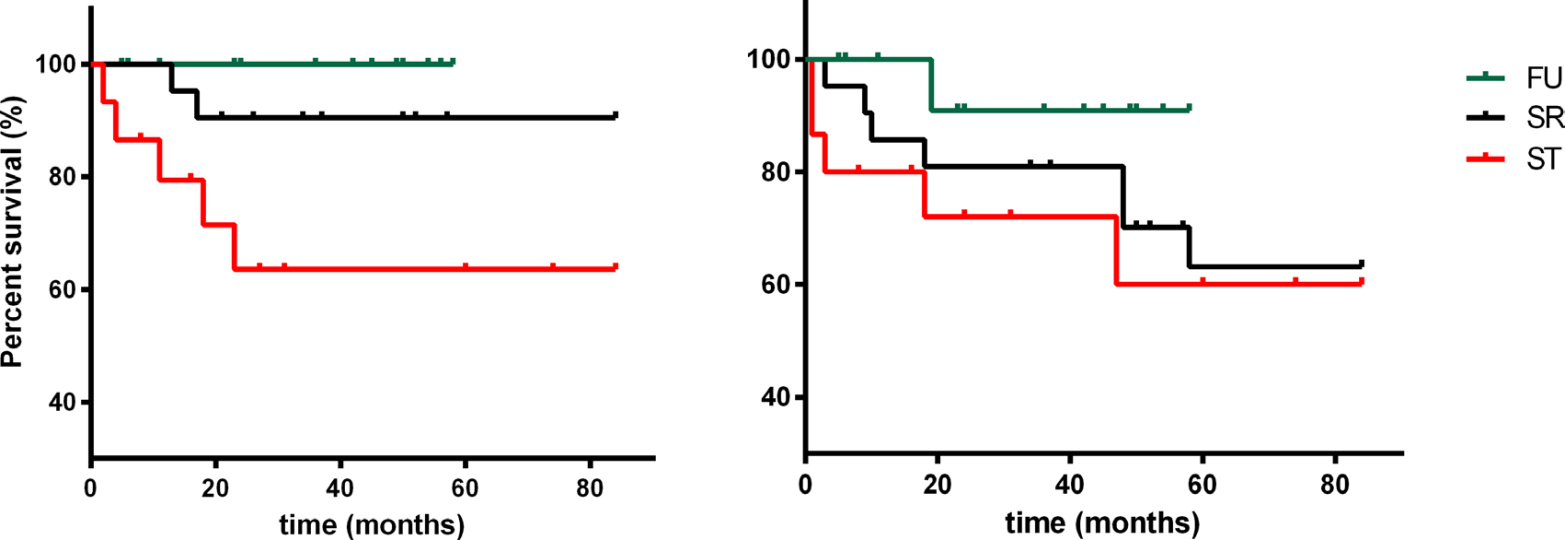
Overall survival (OS) and progression-free survival (PFS) of patients who have undergone follow-up (FU), surgical resection (SR) and systemic therapy (ST), respectively

The OS was similar in the FU pNEN and SR pNEN groups, but, as expected, it was significantly worse in the ST pNEN group (log-rank test p=0.018 for the overall comparison; p=0.023 for the comparison between the ST pNEN and the FU pNEN groups, adjusted for multiple comparisons) (Figure 1). The 4-year survival rate was 100% in the FU pNEN group, 90.5 % in the SR pNEN group and 61% in the ST pNEN one, respectively (p <0.0001 at χ^2^ test).

Progression-free survival (PFS) did not differ among the three groups (log-rank test p=0.304), even if disease remained stable in all but one FU patients (7%), whereas six patients (28%) in the SR group and five (31%) in the ST group showed disease progression after 51 months follow-up.

With regard to the FU pNEN group, the patient with disease progression was a female with a MEN-1 syndrome and showed evidence of lung and nodal metastases during follow-up, 19 months after the initial diagnosis; no surgical therapy was planned after the evidence of disease progression and she underwent medical therapy by somatostatin analogues (SSAs) with disease stabilization.

Among the 21 patients of SR pNEN group we observed a nearly 50% of complications: 5 mild (post-surgical infection), 3 moderate (pancreatic fistula) and 2 severe (severe hemorrhage) events; 6 patients developed post-surgical endocrine insufficiency (i.e. diabetes). None died because of surgical intervention. In this group, 15 patients (71%) showed no disease recurrence, whereas 6 patients (28%) presented disease progression after a median of 14 months (range 3-58) after surgical resection: one had pancreatic recurrence and 5 (83%) had hepatic metastatization. All the 6 patients with progressive disease underwent SSAs therapy; one patient was subsequently administered everolimus therapy, one patient underwent peptide receptor radionuclide therapy (PRRT) and one underwent percutaneous radiofrequency ablation of liver metastases because of further progression disease.

Regarding the ST pNEN group, 5 patients (31%) showed disease progression, after a median of 3 months (range 1–47) after the first diagnosis. All the patients were administered second-line medical therapy, 2 patients were treated with PRRT. Four patients died from disease-related causes.

## DISCUSSION

The present prospective series showed that in terms of both OS and PFS the “wait and watch” approach appears to be rational and safe when dealing with early-stage low-grade ø ≤ 20 mm sized well-differentiated pNENs, although disease progression may occur also in this subset of patients. The optimal management option for small (ø ≤20 mm) non-functioning pNENs remains to be defined. Based on several retrospective series, the ENETS guidelines now recommend a ‘wait and see’ policy for selected patients with small asymptomatic pNENs in view of their slow growth and good overall survival.

Our prospective study generally confirms the opportunity of a conservative approach in these small non-functioning pNENs. In fact, both OS and PFS were similar in the SR pNEN and FU pNEN groups and only one patient experienced disease progression in the FU group after 20 months from the initial diagnosis. That patient, with MEN-1 syndrome, was initially diagnosed with multiple non-functioning pNENs, the largest being ø 13 mm sized. During the follow-up period she developed local progression, nodal and distant metastases, which controindicated a surgical approach; she was treated with SSAs and is currently alive, with stable disease. The disease progression was likely to result from the impredictable biological behavior which characterizes pNENs. The woman was included in the FU pNEN group in view of her tumor features (non-functioning tumor, ø < 2 cm, grade 1) and because the current management policy for MEN-1 syndrome suggests a non-operative approach [18–20]. In addition, for MEN-1 patients the risk of malignancy correlates to the tumor diameter and increases substantially when its size approaches or exceeds ø 3 cm [19, 25].

The inclusion of MEN-1 patients which may be considered a potentially confounding variable reflects our real-life clinical practice. In addition, the distribution of MEN-1 cases is homogeneous among the three patient groups, thus ruling out any effect on overall results.

Moreover, it should be considered that also the patients surgically treated may experience disease progression, as occurred in 6 patients of the present series (3 of them died because of the disease).

Some factors, more recently described but not analyzed in the current series, could be potentially predictive of progression, such as the FDG PET status of pancreatic lesions [26–27]. In fact, the possible selection on the basis of FDG PET would help turning patients to surgery rather than to simple follow-up. Noteworthy, the identification of prognostic factors of tumor growth would be of great help to select for observation only those patients with benign and slow-growing tumors.

The strengths of present study include: its prospective nature which is of relevance as pertinent data from randomized controlled trials are not available, the patients' homogeneous management and follow-up at a single treatment centre, and the inclusion of PFS among the primary endpoints, besides OS, which is clinically more relevant as NENs are known for their generally good prognosis. Conversely, our study presents some limits, in particular, regarding the small sample size and the different tumor stages at presentation of the three groups (Table 1), which may affect the results as the three compared groups presented different prognostic features, i.e. tumor stage and size. In particular an imbalance of prognostic patient characteristics is mainly evident among FU and SR /ST group. Furthermore, the similar survival of FU and SR group may be partially due to the less favorable prognostic features of SR group compared to FU group. However, the study design (i.e. prospective observational study) entails intrinsic limitations which could have been avoided only through a randomized trial, equally distributing the potentially confounding variables among the different groups. Anyway, such study is difficult to be designed due to both the rarity of the disease and the ethic-related issues. Meanwhile, our observational study, despite its intrinsic limitations, may provide preliminary information to support the “wait and see” policy, which is currently suggested by international guidelines and expert opinions [17], even if not properly evidence-based.

**Table 1:** Demographic and laboratory characteristics of patients with pancreatic neuroendocrine neoplasms (pNENs)

Parameter	FU (n=15)	SR (n=21)	ST (n=15)
**Male/Female (n)**	6/9	7/14	7/8
**Age, no. of years**	65 (27-84)	52 (27-82)	72 (27-87)
**TNM stage (no. of pts)**			
**I**	14	2	-
**II**	1	9	7
**III**	-	1	-
**IV**	-	9	8
**Size (mm)**	11 (7-20)	22 (8-60)	26 (20-60)
**Grade (G1/G2/G3)**	15/0/0	10/10/1	8/5/2
**Functioning yes/no (no. of pts)**	-/15	6/15	5/10
**MEN1 (no. of cases)**	3	5	2
**Follow-up (no. of months)**	36 (5-58)	57 (13-84)	27 (2-84)
**Chromogranin A (U/L)**	40 (12-267)	28 (9-3240)	136 (19-1236)

Data are expressed as median (and range) unless specified otherwise.

G1: neuroendocrine tumor with Ki-67 ≤2%; G2: neuroendocrine tumor with Ki-67 between 3 and 20%; G3: NEN with Ki-67 >20%

On the other hand, the comparison between SR and ST groups (well balanced per stage and T size), show a better outcome in SR group respect to ST, confirming the positive impact of primary tumour resection on overall survival in patient in stage IV. Moreover in the FU pNEN group, the staging assessment was performed only through morphological and functional imaging, differently from the patients undergoing surgery, for which the staging was also post-surgical. In the FU pNEN group nodal involvement and/or micrometastases might have also been present and not detected by imaging techniques: this supporting the possible underestimation of disease stage in this cohort, even if in this study a worsening survival has not been observed with regard to those patients treated with a conservative approach.

Despite the reported limitations, we indeed observed that pNENs ø ≤20mm included in the FU group showed a 4-year survival rate of 100%, which represents the most significant result, demonstrating the safety of such an approach.

In summary, on the basis of the present series, consideration should be given to a “wait and watch” approach to carefully selected small early-stage well-differentiated non-functioning pNENs, although further studies comparing patients with the same prognostic characteristics in larger cohort of patients with longer follow-up periods are required in order to confirm these results.

## PATIENTS AND METHODS

Between December 2007 and December 2014, 51 patients were newly diagnosed with pNENs based on clinical data, imaging (computed tomography=CT, magnetic resonance imaging= MRI, ^68^Gallium positron emission tomography= Ga-68 PET), ultrasound endoscopy, histology. The patients had been consecutively enrolled at the Gastroenterology and Endoscopy Unit of Fondazione IRCCS Ca' Granda Ospedale Maggiore Policlinico of Milan, Italy.

The patient characteristics are detailed in Table 1. Eleven cases (21.6%) presented a functioning tumor: 6 with gastrinoma, 2 with glucagonoma, one with insulinoma, one with VIPoma and one had a pancreatic tumor producing serotonin; the remaining 40 patients (78.4%) had a non-functioning neoplasm. The tumors were staged according to the TMN stage scoring system [22] and classified, on the basis of their immunohistochemical characteristics according to the WHO 2010 classification, as pNENs of grade (G)1 (Ki-67 ≤2%), G2 (Ki-67 3–20%) and G3 (Ki-67 >20%) [23].

Chromogranin A (CgA) and specific circulating peptides were evaluated at diagnosis and during follow-up. CgA was measured using a commercially available kit (Dako Chromogranin A Elisa Kit, Dako A/S, Glostrup, Denmark). A regular clinical, biochemical and imaging follow-up was undertaken in all the cases (every quarter during the first year and twice a year thereafter). Morphological imaging was used to evaluate the objective responses (i.e. tumor size) according to the criteria released by the Italian Trials in Medical Oncology group [24]: classifying as complete, partial (with a tumor size decrease of >50%), stable (with a decrease of <50% or an increase 25%) and progressive (if the increase was >25%).

Among the 51 patients, 15 (male/female 6/9, median age 65 years, range 27–84 years) with pNENs ø ≤20 mm were proposed for an intensive 3-month follow-up during the first year and then on 6-month intervals by CT or MRI and every 2 years by Ga-68 PET (FU pNEN group). They were all at stage I according to the TNM classification, except for one patient (stage IIA). All the patients had a well differentiated G1 pNEN. Three patients had a MEN-1 syndrome.

21 patients (male/female 7/14, median age 52 years, range 27–82 years) underwent surgical resection (SR pNEN group) because of functioning neoplasms, grade >G1, size ø >20 mm. Two patients were at TNM stage I, 9 at stage IIA, one at stage IIIB and 9 at stage IV. Ten patients had a G1 pNEN, 10 had a G2 pNEN and one had a G3 pNEN. Six patients (28.6 %) had a functioning tumor; five had a MEN-1 syndrome.

The remaining 15 patients (male/female 7/8, median age 72 years, range 27–87 years) received systemic therapy (ST pNEN group) because of their advanced disease or contraindications to surgery; 5 patients were at stage IIA, 2 at stage IIB, 8 at stage IV. Eight patients had G1, 5 had G2 and 2 had G3 pNEN. Five patients had a functioning tumor, 2 had a MEN-1 syndrome.

All the patients gave their written informed consent to the study, which had been approved by the local Ethics Committee.

### Statistical analysis

All the continuous variables are reported as median and range unless stated otherwise. Continuous data were analyzed using the non-parametric Mann-Whitney test. Differences between percentages were evaluated by χ2 test.

The survival curves were estimated using the Kaplan-Meier method and the log-rank test was used to compare the survival curves between different patient groups.

Statistical analyses were performed using MedCalc sotware and a p value less than 0.05 was considered statistically significant.
